# Tanzanian Couples' Perspectives on Gender Equity, Relationship Power, and Intimate Partner Violence: Findings from the RESPECT Study

**DOI:** 10.1155/2012/187890

**Published:** 2012-12-24

**Authors:** Suneeta Krishnan, Divya Vohra, Damien de Walque, Carol Medlin, Rose Nathan, William H. Dow

**Affiliations:** ^1^RTI International, 114 Sansome Street, Suite 500, San Francisco, CA 94104, USA; ^2^Division of Epidemiology and Biostatistics, University of California Berkeley, 101 Haviland Hall, Berkeley, CA 94704, USA; ^3^Development Research Group, The World Bank, 1818 H Street NW, Washington, DC 20433, USA; ^4^Health Economics and Finance, Global Health Program, The Bill and Melinda Gates Foundation, P.O. Box 23350 Seattle, WA 98102, USA; ^5^Ifakara Health Institute, Plot 463 Kiko Avenue, Mikocheni, Dar-es-Salaam, Tanzania; ^6^Division of Health Policy and Management, University of California Berkeley, 239 University Hall, Berkeley, CA 94704, USA

## Abstract

Intimate partner violence (IPV) is widely prevalent in Tanzania. Inequitable gender norms manifest in men's and women's attitudes about power and decision making in intimate relationships and are likely to play an important role in determining the prevalence of IPV. We used data from the RESPECT study, a randomized controlled trial that evaluated an intervention to prevent sexually transmitted infections in a cohort of young Tanzanian men and women, to examine the relationship between couples' attitudes about IPV, relationship power, and sexual decision making, concordance on these issues, and women's reports of IPV over 12 months. Women expressed less equitable attitudes than men at baseline. Over time, participants' attitudes tended to become more equitable and women's reports of IPV declined substantially. Multivariable logistic regression analyses suggested that inequitable attitudes and couple discordance were associated with higher risk of IPV. Our findings point to the need for a better understanding of the role that perceived or actual imbalances in relationship power have in heightening IPV risk. The decline in women's reports of IPV and the trend towards gender-equitable attitudes indicate that concerted efforts to reduce IPV and promote gender equity have the potential to make a positive difference in the relatively short term.

## 1. Introduction

Intimate partner violence (IPV) is a major public health and human rights concern in Tanzania [1]. According to the World Health Organization's Multi-country Study on Women's Health and Domestic Violence (2000–2003), the prevalence of physical and/or sexual IPV was 41% and 56%, respectively, among a representative sample of ever-partnered women in Dar es Salaam and the southern district of Mbeya [2]. A growing number of studies have documented the association between IPV and an array of adverse reproductive and sexual health outcomes, including pregnancy loss and HIV infection among women in Tanzania [3–7]. Of particular concern is evidence on the links between IPV, sexual risk behaviors, and HIV infection among young Tanzanian women and men [6–9]. According to one study at an HIV voluntary counseling and testing center in Dar-es-Salaam, the odds of IPV were ten times higher among young HIV-positive women (<30 years of age) than among similarly aged HIV-negative women [6]. Another study involving 951 young men aged 16 to 24 years in two neighborhoods of Dar-es-Salaam found that about a third had ever perpetrated IPV and that those who reported more lifetime sexual partners were also more likely to perpetrate IPV [7]. Given the high prevalence of IPV and its adverse health impacts, a better understanding of the risk of IPV, especially among young women, is needed.

It is widely accepted that gender inequities, perpetuated by cultural norms regarding gender roles and manifest in men's and women's ideas about power and decision making in relationships, have a profound impact on the perpetration and experience of IPV [10]. Several qualitative studies in Tanzania have documented the links between entrenched gender inequities and IPV [8, 9, 11]. In one study, 16–24-year-old men and women in Dar-es-Salaam described the ideal woman as one who is home-bound, loyal to her partner, and sexually submissive [9]. Young women who deviated from these prescribed behaviors risked being beaten. Infidelity or perceptions of infidelity were the most commonly cited triggers of violence against female partners across studies [8, 9]. Men and women often justified violence against a female partner as a response to a woman's infidelity or confrontations regarding a man's infidelities. Furthermore, it was not uncommon for women to be blamed for provoking IPV, preventing women from seeking support or medical care, and making law enforcement difficult [12].

Survey data lend support to the observation that both men and women in Tanzania condone IPV as a normal part of an intimate relationship [7, 13]. According to the Demographic and Health Survey (DHS, 2004), 60% and 42%, respectively, of women and men found spousal abuse to be acceptable under one or more scenarios (e.g., wife neglects child, goes out without permission, argues with husband, etc.) [13]. Maman et al. reported that 46% of a sample of young men condoned violence against a female partner in one or more circumstances [7]. A similar proportion of women attending an HIV voluntary counseling and testing center in Dar-es-Salaam felt that physical abuse was justified in at least one of several situations such as infidelity, disobedience, and nonperformance of domestic work [14]. 

Although research has explored men's and women's attitudes about IPV, few studies have empirically examined the association between these attitudes and IPV risk [15]. For example, a cross-sectional survey of men working in three municipalities in Cape Town, South Africa found that men who thought it was acceptable to hit women were more likely to also report recent or past physical violence against a partner [16]. Still fewer studies have assessed the relationships between concordant or discordant attitudes towards IPV within a couple and women's experience of IPV. A recent analysis of DHS data from six African countries (Kenya, Liberia, Malawi, Rwanda, Zambia, and Zimbabwe) examined the relationship between couple concordance on attitudes towards IPV (partner agreement that violence is justified in at least one situation) and IPV (any physical or sexual violence reported by women) [17]. The authors found that IPV was more commonly reported among couples who agreed that IPV was acceptable in at least some situations as well as those who expressed discordant attitudes towards IPV compared to couples who agreed that IPV was never acceptable. Notably, statistically significant associations between concordance on IPV acceptability and reported IPV and between discordance and IPV were observed in five and four out of six countries, respectively. 

To our knowledge, the association between couples' attitudes towards IPV, couple concordance in attitudes and IPV risk has not been examined in Tanzania. Using data from the Rewarding STI Prevention and Control in Tanzania (RESPECT) study, we examined men's and women's attitudes about IPV, relationship power, and sexual decision making and couples' concordance on these issues, and whether these attitudes were associated with women's experience of IPV at baseline and over time. 

## 2. Methods

### 2.1. The RESPECT Study

The year-long RESPECT study was a randomized controlled trial designed to evaluate whether conditional cash transfers (CCT) promoted safe sex and reduced the incidence of sexually transmitted infections (STIs) (see [18] for additional details regarding the study). Women and men aged 18–30 years living in 10 villages in the Kilombero/Ulanga districts in south-western Tanzania were randomly selected from the Ifakara Demographic and Health Surveillance System database. Participants who were interested in enrolling jointly with their spouse were encouraged to do so and considered to be a couple if they each reported that they were married to one another or were living together as if married. Couples were linked through a common household identification number. 

About 50% of participants were randomly assigned to a no-payment control group, 25% to a low-value CCT group, and the remaining 25% to a high-value CCT group. Participants were followed for 12 months and interviewed every 4 months to gather data on a range of issues, including sociodemographic background, economic status, sexual and reproductive health knowledge, practices, and history, attitudes about IPV and relationship power, as well as experiences of IPV (women) and perpetration of IPV (men). They also underwent STI and HIV counseling and testing. Participants in the CCT arms received cash payments for every 4 monthly negative STI laboratory test result. All enrolled individuals were invited to group counseling sessions that focused on relationship and life skills training based on the Stepping Stones curriculum [19]. 

### 2.2. Theoretical Framework and Hypotheses

The analysis is guided by a social-ecological framework, which posits that IPV risk is shaped by the interplay of a host of individual, community, and societal factors, including individual beliefs and practices within an intimate relationship as well as community and societal norms regarding gender and power [20]. It is also informed by the proximate determinants framework proposed by Boerma and Weir, which enables the classification of factors into distal and proximate predictors of IPV [21]. According to the proximate determinants framework, ecological factors such as cultural norms influence a particular health outcome through a set of intermediate or proximate variables. These proximate determinants, which can include a combination of social and biological factors, directly influence the health outcome of interest. 

In this analysis, we considered attitudes toward IPV as proximate determinants, and gender norms as a key underlying, distal determinant of IPV. For example, women's access to education and employment is limited in social and cultural environments that are highly patriarchal, increasing their economic dependence on male partners, which is a known risk factor for IPV (i.e., a proximate determinant) [22]. Similarly, inequitable gender norms can also create an environment that is generally tolerant of male dominance in intimate relationships and violence against women (a distal determinant) [23], and influence both men's and women's attitudes towards IPV (a proximate determinant), and in turn affect women's experience of IPV. 

We outlined our hypotheses about the causal relationships between all variables in a Directed Acyclic Graph (DAG; not presented) and used the DAG to determine the minimum variables necessary to include in multivariable analyses to remove confounding of the main effects. Our primary hypothesis is that men's and women's attitudes about IPV, relationship power, and sexual decision making (including couple concordance/discordance on these attitudes) are proximate determinants of women's experience of IPV. Specifically, we proposed that women's and men's espousal of inequitable gender attitudes would be associated with greater experience of IPV at baseline and over time. We also hypothesized that couples' discordance on these issues would be associated with a heightened risk of IPV at baseline, and that this relationship would persist over time. We further hypothesized that the very fact of discordance between couples is more important than the nature of that discordance; that is, we proposed that lack of agreement between a woman and her partner (regardless of which partner held the more inequitable attitudes) would be associated with a higher risk of IPV than if she agreed with her partner. This finding would be consistent with earlier studies that have found that women themselves often exhibit highly inequitable attitudes about IPV as a way of fitting in with their communities and protecting themselves from violence [24, 25]. 

### 2.3. Ethical Considerations

Study protocols were approved by institutional review boards in Tanzania and the United States. All study participants gave written informed consent to participate in the study. Couples were interviewed separately at a study station that was set up on the outskirts of the village, and care was taken to ensure privacy and confidentiality. Study interviewers received in-depth training on interviewing techniques, gender and reproductive health, and the study protocols. A study liaison was identified in each village to help participants gain access to further information, counseling services, and study personnel. In addition, study counselors received training on how to offer psychosocial support and were equipped with information on domestic violence-related services.

### 2.4. Measures

The outcome of interest—women's self-report of intimate partner violence over the previous 4-month period—was measured using a dichotomous variable based on four questions from the RESPECT questionnaire: “have you been hit, kicked, or beaten by your partner and/or a family member for any reason during the last 4 months?”, “Has your partner or another family member done any of the following during the last 4 months: humiliated you in front of others, insulted you, tried to scare you, threatened to hurt you or someone you care about?”, “Have you been physically forced to have sexual intercourse when you did not want to during the last 4 months?”, and “Did you, during the last 4 months, have sexual intercourse when you did not want to because you were afraid of what your partner might do?”. Participants who responded “yes” to one or more of these questions were coded as having experienced violence, while those who responded “no” to all four questions were coded as not having experienced violence. The RESPECT questionnaire did not ask women about lifetime experience of violence; at all rounds, women were asked about their experience of violence in the previous four months. 

Although the RESPECT questionnaire asked similar questions regarding male participants' perpetration of violence against their partners, couples did not always agree on violence within their relationships (data not shown). Given this disagreement and our primary interest in examining women's experience of IPV during the course of the study, we decided to focus on women's report of violence as the outcome measure. 

Our analyses focused on the association between women's reports of IPV and women's and men's attitudes about IPV, relationship power, and sexual decision making and couples' concordance. Men's and women's attitudes towards IPV and opinions about power within relationships were assessed using four exposure variables. The first question (“is a husband justified in beating his wife if…”) measured the acceptability of physical IPV in five hypothetical situations: if a wife goes out without telling her husband, if she neglects the children, if she argues with her husband, if she refuses to have sex with her husband, or if she burns the food. A binary variable was created to measure acceptability of IPV, coded as “1” if a participant responded in the affirmative to any of these five situations and coded as “0” if the participant did not agree that violence was justified in any of these situations. The second question assessed the acceptability of IPV as a response to a wife refusing to have sex with her husband: “if a woman refuses to have sex with her husband when he wants her to, he has the right to: get angry and reprimand her, refuse to give her money or other means of financial support, use force and have sex with her even if she doesn't want to, or go and have sex with another woman.” A binary variable (yes/no) was created on the basis of whether participants thought IPV was acceptable in response to a wife's refusal to have sex. For each of these questions, couples were coded as having concordant responses if both partners shared the same binary response.

Our third and fourth exposure variables of interest assessed participants' opinions about power within their relationship. These were ascertained using the following two questions: “who usually has more say about whether you have sex?” and “in general, who do you think has more power in your relationship?” Participants were given the response options “myself”, “my partner”, or “both people equally.” Couples were determined to be concordant if they shared the same response about which partner had more to say about having sex or had more power in the relationship, regardless of which partner this was or whether they agreed that they shared these decisions equally. 

Other covariables we considered included age (measured as a continuous variable), education status (measured as a categorical variable—no schooling, some primary school, primary school completed, some secondary school, secondary school completed, and postsecondary or university education), and socioeconomic position (measured by asking participants to rate themselves on a scale from 1 to 7 relative to others in their community). We also examined differences in reported IPV by study arm.

### 2.5. Statistical Analyses

Analyses were conducted using data from the subset of heterosexual couples who were enrolled in the study together. All couples were included in the baseline data analysis, and couples on whom data were available for a minimum of two out of the four rounds were included in the longitudinal analyses. For each round, couples were included in the analysis as long as there were no missing data on the variables of interest. 

Preliminary analyses focused on the cross-sectional relationships between age, education, and socioeconomic position and ever having experienced IPV at baseline using contingency tables, Chi-square analyses, and Student's *t*-tests. Next, we looked at changes in women's reports of IPV as well as changes in participant's attitudes about IPV and relationship power during the follow-up period. We conducted tests for trend to determine whether changes were statistically significant. 

To examine the independent relationship between the exposure variables (women's and men's attitudes towards IPV and relationship power and partner concordance on attitudes) and IPV, we fit separate logistic regression models for each indicator. We also ran a multivariable logistic regression model to examine the association of each main exposure variable and IPV, adjusting for socioeconomic status, age, and education. 

Finally, to examine the longitudinal relationship between the exposure variables of interest and women's experience of IPV, we used multivariable random effects logistic regression models [26]. These models were used to examine the effect of changes in men's and women's attitudes about IPV and relationship power and couple concordance on odds of experiencing IPV over time. A random effects model was chosen to evaluate the change in IPV odds for a single woman when she expressed inequitable attitudes versus when she expressed equitable attitudes. This model produced an odds ratio of experiencing IPV for an individual woman when she expressed inequitable attitudes relative to when she expressed equitable attitudes. Similar interpretations apply to the set of analyses run on men's attitudes, as well as the set of analyses on couple concordance. Clustered standard errors were used to account for the nonindependence of an individual's observations over time.

Socioeconomic position, age, education, and round of data collection were included in the models as confounders. Interactions between these confounders and the exposures of interest were also considered. However because they were not statistically significant, they were not included in the final model. 

We examined three “families” of hypotheses: based on women's attitudes and opinions, men's attitudes and opinions, and couple concordance. We believed that an individual hypothesis within each family would have to be considered in light of the additional tests performed on other hypotheses within the subgroup. Since each family of hypotheses included four exposure variables, we determined that an appropriate significance level (alpha) for each hypothesis test would be set at 0.05/4 or 0.0125. 

## 3. Results

Out of a total of 2,399 individuals enrolled in RESPECT, 567 couples were identified and included in this analysis. A comparison of individuals who reported being married or living together as married and who did not enroll as a couple and those who did enroll as a couple indicated that there were no statistically significant demographic differences between the two groups. A total of 26 couples were lost to follow up: seven after the baseline round and an additional 19 between rounds 2 and 4. Additionally, at each round, between two and four couples were missing data on one or more variables and were excluded from the analysis. Couples who were lost to follow or excluded due to missing data did not differ in terms of demographic characteristics or women's reports of IPV (data not shown). 

Participant characteristics at baseline including demographic background, experiences of IPV, attitudes about violence and opinions about sexual decision making and relationship power are shown in Table 1. About one in five women (20.5%) reported experiencing IPV at baseline. Women who reported experiencing IPV and those who did not were of similar age and had similar levels of education and self-reported socioeconomic status. Of note is the fact that large proportions of men and women felt that IPV was justified in some instances: at baseline, 71% of women and 48% of men reported that beating a wife was justified in one or more situations. In addition, according to both women and men, husbands had more say over sex and had more power in their relationship than wives. Overall, men espoused more gender-equitable attitudes than women.

Analyses revealed that women's reports of violence and participants' attitudes about IPV and opinions about sexual decision making and relationship power changed consistently and substantially over the 12-month follow-up period. Reported IPV (in the four months prior to the interview) decreased steadily over time from 20.5% at baseline to 11.8% at 12 months (data not shown). The decrease was statistically significant (*P* < 0.0005) and was not associated with demographic characteristics or study arm. In addition, at 12 months, fewer women and men noted that violence against a wife was acceptable, and a larger proportion of participants reported that sexual decision making was shared by both partners (Table 2). Interestingly, for both men and women, responses to questions about the acceptability of IPV showed more dramatic changes from baseline to 12 months than responses to questions about power within relationships, which barely changed. No changes in the level of couple discordance/concordance in attitudes about IPV were observed (data not shown).

Table 3 summarizes the results of the longitudinal and random effects of multivariable logistic regression analyses. The associations between men's attitudes about IPV and relationship power and spousal reports of IPV were in the hypothesized direction, but were not statistically significant. However, several measures of women's attitudes about IPV and relationship power were statistically associated with their reports of IPV. Women who reported that violence was ever justified if a woman refuses sex were more than twice as likely to report IPV (adjusted OR = 2.29, 95% CI: 1.65–3.17). Furthermore, women were less likely to report IPV when they said that both partners shared sexual decision making (adjusted OR = 0.70, 95% CI: 0.5–0.98), as compared to women who said that their partner controlled sexual decision making. Notably, we found that women were less likely to report IPV when they said that both partners had equal power (adjusted OR = 0.43, 95% CI: 0.21–0.89) or that they controlled more power (adjusted OR = 0.91, 95% CI: 0.28–2.94). For all four exposures of interest, women were more likely to report IPV when couples expressed discordant attitudes relative to when they shared concordant attitudes, but these effects were relatively small and not statistically significant (Table 3). 

In all longitudinal analyses, a statistically significant portion of the variance of the estimates was due to the random effect of individuals, suggesting that there was a significant amount of between-subject variation (data not shown). 

## 4. Discussion

This longitudinal analysis suggests that couples' attitudes towards violence and opinions about sexual decision making and relationship power are proximate determinants of women's experience of IPV. The study also provides some evidence that discordance among couples on these issues may heighten women's risk of experiencing IPV. 

Our observation that gender inequitable attitudes were more commonly reported by women than men is consistent with findings from other studies [13]. It is possible that due to social desirability bias, men were less likely than women to openly agree that violence against women is justified. However, researchers have suggested that women's acceptance of IPV and conformity to dominant understandings of gender roles and relationships is likely to be an expression of their experience and expectations as well as a reflection of prevailing social norms [11, 24, 25]. Studies elsewhere in the world have noted that women who transgress norms, for example, by choosing their spouse or by seeking economic independence, are more likely to experience IPV [27, 28]. Indeed, conformity to social norms and expectations may be a protective mechanism-enabling women to fit in and avoid family and community censure. Qualitative research in Tanzania suggests that pressures on women to conform are considerable. In Lary et al.'s study in Dar-es-Salaam, young female participants placed “great value on community perceptions of their character” and noted that even taking a walk may raise family and community members' suspicions [9]. Other research by Laisser et al. in an urban community in Tanzania also highlighted women's internalization of inequitable norms. In the words of one female participant, “we annoy our husbands with our behaviours and sometimes we deserve to be beaten [11, page 5].” That said, the authors also noted that perceptions of IPV may be changing, with both men and women acknowledging the adverse impacts of violence on women's self-esteem, health, and dignity and expressing a need for governmental action, including laws against IPV and health care services for survivors [11].

It is encouraging to note that men and women tended to express more gender equitable attitudes by the end of the study. The fact that attitudes about the acceptability of IPV changed far more than opinions about sexual decision making and power within participants' relationships suggests that the changes could have been partly a result of social desirability bias. However, reported 1PV declined steadily over the course of the study from 20% at baseline to 12% at the end of one year of followup, and were not associated with demographic characteristics or intervention/control status. Since we have data on levels of IPV only from RESPECT study participants, we cannot determine whether this result reflects a declining trend in IPV in this region. However, to our knowledge, no major interventions on IPV occurred during this time period and it is unlikely that such a substantial reduction in IPV could be explained in this fashion. The reduction in women's reports of IPV—despite improved rapport between participants and study staff (which may have improved IPV disclosure)—suggests that changes in men's attitudes and behaviors may have resulted from study participation. Given that the proportion of individuals who participated in the group counseling sessions on relationship and life skills was low (data not shown), and that STI/HIV counseling did not explicitly address relationship issues, we hypothesize that repeated exposure to survey questions on relationship dynamics and the opportunity to participate in the study as a couple may have contributed to these shifts. Engaging men and women—as individuals, couples, and community members—is widely accepted as an important component of IPV prevention efforts worldwide [23, 29]. At a minimum, our study demonstrates the feasibility, safety, and potential effectiveness of engaging young Tanzanian men and women as couples in programs that address subjects considered controversial or taboo in their communities.

Results of the longitudinal regression analyses point to the potential benefits of promoting notions of equity in relationships. Women who reported that they shared sexual decision making and relationship power with their partner were consistently less likely to report IPV. In contrast, IPV was reported more frequently when men and women espoused inequitable attitudes or reported that women had more decision making control in the relationship although few of these associations were statistically significant. These findings underscore the need to better understand the delicate balance of power in intimate relationships and the role that perceived or actual imbalances in power (especially in favor of women) have in heightening women's risk of IPV. Further qualitative research may shed light on the dynamics of power, conflict, and violence within relationships in which partners hold similar or differing views.

The association between couples' concordance on attitudes about IPV and relationship power and women's experience of violence also merits further investigation. Our study had limited statistical power to investigate the relationship between different types of concordance/discordance and IPV risk. Thus, we were unable to examine whether IPV risk differed depending on who held more equitable attitudes within a relationship. For example, future research should explore whether risk is higher among women who feel IPV is unjustified and whose partners feel it is justified. Previous research has suggested that discordance within a couple arising from perceived or actual gains in power by women can result in backlash, including IPV by men [27, 29, 30]. However, researchers have also pointed out that women can also be resistant to changes in gender roles and relations and unwilling to let go of their beliefs and expectations regarding men's and women's roles and responsibilities within relationships, leading to conflict and violence [29]. 

Overall, much remains to be learned about how women and men perceive and engage with ideas of greater equity in intimate relationships. Gender norms and values are dynamic, and their relationship with individual behaviors and experiences is complex. Further in-depth examination of young women's and men's evolving ideas about gender, identity, and relationships is needed. Several questions merit study. For example, do young men and women perceive their relationship to be “healthy”? Do they desire greater equity and how do they define equity in a relationship? Are these views—and concordance/discordance in views within a couple—associated with how partners communicate with each other, handle conflicts, and experience or perpetrate IPV? A better understanding of these questions will further illuminate the ways in which gender norms and relationship dynamics influence women's risk of experiencing of violence and help identify entry points for IPV prevention efforts.

Our study has additional limitations. First, the decision to measure IPV as a binary variable without accounting for frequency or type of IPV, while providing us with more statistical power, may have prevented us from observing crucial differences in the associations between attitudes and IPV risk. Second, it is especially difficult to draw strong conclusions about the heightened risk of IPV among couples holding discordant attitudes without a finer understanding of how the composition of this discordance might differently impact women's experience of IPV. Third, the decision to use only partnered couples in these analyses also raises issues of potential selection bias. It is possible that partners who both chose to participate in the RESPECT study differed in important ways from participants whose partners chose not to be in the study, including on attitudes about the acceptability of IPV. Finally, it is possible that women who experience IPV are more likely to report that violence is justified. 

## 5. Conclusions

Despite its limitations, this research provides some new insights on the role of women's and men's attitudes toward IPV and relationship power, including the role of partner discordance, in influencing women's experience of IPV. Unlike most previous research in Tanzania, this study prospectively examined the relationship between attitudes about gender relations and IPV among young couples. The widespread acceptance of IPV and inequitable power within relationships in this population highlights the urgent need for programs that help young people acknowledge, understand and challenge gender-based hierarchies. Greater understanding of young people's perceptions of “gender equity”—by focusing on women and men who do not condone IPV and who share power within their relationship—will facilitate the development of antiviolence programs. Furthermore, couple-based programs for HIV testing and treatment have been successful in sub-Saharan Africa and offer a foundation for antiviolence efforts [29]. The decline in women's reports of IPV and the trend towards gender-equitable attitudes that we observed in the RESPECT study indicate that concerted efforts to reduce IPV and promote gender equity have the potential to make a positive difference in the relatively short term.

## Figures and Tables

**Table 1:** Baseline characteristics of couples in the RESPECT study.

			Women					
Variable	Men						All women	
			IPV		No IPV			
*N* ^a^	567		114		442		567	
Mean age (min, max)	32.9 (19, 60)		26.3 (18, 35)		26.6 (17, 35)		26.5 (17, 35)	

	*n*	%	*n*	%	*n*	%	*n*	%

Education status (%)								
None	43	7.7	18	15.8	63	14.3	82	14.5
Some primary	87	15.5	32	28.1	108	24.4	142	25.4
Primary completed	389	69.3	60	52.6	258	58.4	319	57.0
Some secondary	23	4.1	2	1.7	9	2.0	11	2.0
Secondary or higher completed	19	3.4	2	1.7	4	0.9	6	1.1
Self-reported SEP (%)								
0–2 (low)	278	49.0	61	53.5	237	53.6	303	53.5
3–7 (high)	289	51.0	53	46.5	205	46.4	263	46.5
Attitudes about IPV								
Is a husband ever justified in beating his wife?								
Yes	265	47.7	84	73.7	308	70.5	394	71.0
No	291	52.3	30	26.3	129	29.5	161	29.0
Is any kind of violence justified if a woman refuses sex?								
Yes	248	44.5	89*	78.8	269*	61.4	360	64.9
No	309	55.5	24*	21.2	169*	38.6	195	35.1
Opinions about relationship power								
Who has more say about having sex?								
Husband	304	54.6	83	72.8	290	65.6	374	67.0
Wife	74	13.3	8	7.0	24	5.4	32	5.7
Both	179	32.1	23	20.2	128	29.0	152	27.3
Who has more power in your relationship?								
Husband	361	65.2	101	88.6	384	86.9	487	87.3
Wife	85	15.3	5	4.4	12	2.7	17	3.0
Both	108	19.5	8	7.0	46	10.4	54	9.7

^a^Distributions of baseline characteristics do not always add up to total *n* because of missing responses.

*P < 0.05.

**Table 2:** Attitudes about IPV and opinions about relationship power at 12 months and changes over time.

	Men's attitudes		Women's attitudes	
	Month 12	Change in percentage points from baseline	Month 12	Change in percentage points from baseline
Is a husband ever justified in beating his wife?				
Yes	144 (26.8%)	−20.9*	313 (57.5%)	−13.5*
No	394 (73.2%)	20.9*	231 (42.5%)	13.5*
Is any kind of violence justified if a woman refuses sex?				
Yes	181 (33.6%)	−10.9*	263 (48.3%)	−16.6*
No	357 (66.4%)	10.9*	281 (51.7%)	16.6*
Who has more say about having sex?				
Husband	259 (48.5%)	−6.1*	292 (53.7%)	−13.3*
Wife	9 (1.7%)	−11.6*	27 (5.0%)	−0.7
Both	266 (49.8%)	17.7*	225 (41.4%)	14.1
Who has more power in your relationship?				
Husband	424 (79.4%)	14.2	486 (90.2%)	2.9
Wife	13 (2.4%)	−12.9	6 (1.1%)	−1.9
Both	97 (18.2%)	−1.3	47 (8.7%)	−1.0

*Statistically significant trend (*P* < 0.05).

**Table 3:** Men's and women's attitudes as predictors of women's IPV report, multivariable logistic regression analysisa.

	Men's attitudes		Women's attitudes		Couple discordance^d^	
	OR	95% CI	OR	95% CI	OR	95% CI
Is a husband ever justified in beating his wife?						
Yes^b^	1.34	0.95, 1.88	1.31	0.93, 1.85	1.01	0.75, 1.37
Is any kind of violence justified if a woman refuses sex?						
Yes^b^	1.06	0.76, 1.47	2.29	1.65, 3.17*	1.35	0.99, 1.79
Who has more say about having sex?						
Wife^c^	1.13	0.60, 2.16	1.31	0.70, 2.45	1.06	0.78, 1.45
Both^c^	0.93	0.66, 1.30	0.70	0.50, 0.98**		
Who has more power in your relationship?						
Wife^c^	1.40	0.73, 2.66	0.91	0.28, 2.94	1.20	0.89, 1.69
Both^c^	1.33	0.84, 2.11	0.43	0.21, 0.89**		

^a^The adjusted model assesses the relationship between each independent variable and IPV, adjusting for the other independent variables in the model and confounders-age, socioeconomic position and education (see Measures).

^b^Reference group is never justified.

^c^Reference group is husband.

^d^Reference group is concordance in responses.

*P < 0.0125.

**P < 0.05.
